# Effects of *Aloe vera* Flower Extract and Its Active Constituent Isoorientin on Skin Moisturization via Regulating Involucrin Expression: In Vitro and Molecular Docking Studies

**DOI:** 10.3390/molecules26092626

**Published:** 2021-04-30

**Authors:** Sultana Razia, Hyunsung Park, Eunju Shin, Kyu-Suk Shim, Eunae Cho, Sun-Yeou Kim

**Affiliations:** 1 Department of Life Science, University of Seoul, Seoul 02504, Korea; raziasultana@pharm.jnu.ac.bd (S.R.); hspark@uos.ac.kr (H.P.); 2 Univera Co., Ltd., Seoul 04782, Korea; ejayshin@univera.com (E.S.); kssim93@univera.com (K.-S.S.); Eunaec@univera.com (E.C.); 3 College of Pharmacy, Gachon University, 191 Hambakmoero, Yeonsu-gu, Incheon 21936, Korea

**Keywords:** *Aloe vera* flower, skin barrier function, involucrin, isoorientin, skin moisturization

## Abstract

Skin moisturization is very crucial for maintaining the flexibility, viscoelasticity, and differentiation of the epidermis and its deprivation causes several diseases from dry skin to dermatitis. *Aloe vera*, a miracle plant having diverse medicinal properties including skin moisturization effects. This study investigated for the first time the molecular mechanism targeting skin moisturization effects of the *Aloe vera* flower and its major active constituent. By treating human epidermal keratinocytes (HaCaT cells) with *Aloe vera* flower water extract (AFWE), we found that AFWE upregulated epidermal involucrin by activating the expression of protein kinase C, p38, and ERK 1/2. Additionally, it modulated filaggrin, increased aquaporin expression, and hyaluronan synthesis via a balanced regulation of HAS1 and HYAL1 protein. Similarly, it was able to protect UVB-induced photodamage. Western blot analysis, ELISA, and qRT- PCR were performed to evaluate various epidermal differentiation markers and moisturization-related factors on human epidermal keratinocytes (HaCaT cells). TLC and HPLC were used to detect and analyze the chemical constituents. Among them, we found that an active component of *Aloe vera* flower, isoorientin (IO) has a high binding affinity to all of its targeted proteins such as involucrin, PKC, P38, etc. through molecular docking assay. This study indicated that the *Aloe vera* flower and its active constituent, IO can be used as a prominent ingredient to enhance skin barrier function and improve its related pathologies.

## 1. Introduction

The skin is the largest external organ of our body with three distinct layers called the epidermis, dermis, and hypodermis. The stratum corneum (SC) of the epidermis acts as the primary defense barrier against physical, chemical, and environmental insults [1,2]. In particular, intracellular lipids and natural moisturizing factor (NMF) of SC help to maintain skin moisturization by preventing trans-epidermal water loss (TEWL) [3,4]. NMF consists of different amino acids and other hygroscopic molecules derived from the proteolysis of filaggrin and other components of the stratum corneum [5]. Impaired SC due to lack of NMF, water, and harsh environmental conditions (UVA or UVB) aggravates dry skin and related several dermatological disorders such as dermatitis, pruritus, psoriasis, inflammation, and aging [4,6].

Based on the known molecular signaling pathways associated with skin moisturization, several components of the epidermal extracellular matrix are reported to be involved in regulating the skin barrier function. A hydrodynamic regulator has a prominent role in keratinocyte regeneration, proliferation, and migration. The amount of hyaluronan is determined by the balance between HA synthases (HASes) and hyaluronidases (HYALs) [7]. It is polymerized by three major HA synthases (HASes) named HAS1, 2, and 3 in humans. On the other hand, its degradation is occurred by various hyaluronidases (HYALs) [8].

Aquaporins (AQP) are the water channels embedded in the plasma membrane dedicated to transport water against the osmotic gradient. Among 13 diverse types of mammalian aquaglyceroporins, aquaglyceroporin (AQP3) is the most prevalent in the skin epidermis [9]. In the granular layer of the epidermis, keratin binding proteins named filaggrin are important contributing factors to maintain epidermal barrier integrity. Profillagrin (PFLG) is cleaved by various proteases to form active filaggrin (FLG). Involucrin (IVL) which is incorporated into the cornified envelope enhances the repairment of skin barrier function and absence of which can be notified with delayed barrier acquisition and hyperkeratotic dry skin with various skin defects [10].

Traditionally, moisturizers from natural sources such as *Aloe vera*, *Centella asiatica*, and seaweed are well-known medicinal plants that have been used to treat several skin diseases [11,12]. To date, *Aloe vera* (L.) Burm. f. (Asphlodelaceae) has been used in cosmeceuticals because of its reported moisturizing, anti-inflammatory, antimicrobial, and wound healing properties, etc. [13]. It is a perennial succulent shrub, with green leaves surrounding the stem in a rose-like pattern, and bicolor flowers grow at the center of the leaves [14]. Orientin (O), isoorientin (IO), vitexin (V), and isovitexin (IV) are C-glycosyl flavonoids that have been reported to present and perform a vital role in this flower [15].

The cosmeceuticals benefits of leaf extract, gel, skin, or their chemical constituents of *Aloe vera* have been extensively investigated in many in-vitro, and in-vivo studies and clinical trials [16]. There are very few studies that have been conducted on *Aloe vera* flowers; however, almost all of them are mainly focused on the identification of chemical constituents of the extracts having antioxidant and antimicrobial properties [17,18]. Therefore, this study has been designed to explore the effect of *Aloe vera* flower on skin moisturization along with the identification of most active constituents based on signaling pathways.

## 2. Results

### 2.1. AFWE Increases IVL Expression as Well as Hyaluronan Secretion by Upregulating HAS 1 and Downregulating HYAL 1 Expression

**Figure 1:**
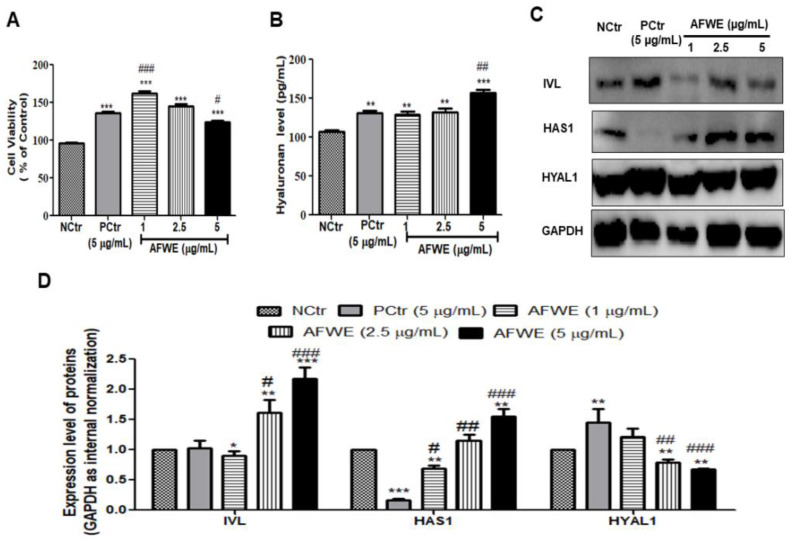
AFWE increases IVL expression as well as hyaluronan secretion by upregulating HAS 1 and downregulating HYAL 1 expression on HaCaT cells. (**A**) Cell viability of AFWE by MTT. (**B**) Hyaluronan level detected by ELISA. (**C**) Western blot analysis of IVL, HAS1, and HYAL1 protein expression. (**D**) Fold change of IVL, HAS1, and HYAL1 expression compared to control group. NCtr is control, PCtr is *C. asiatica*, used as a positive control at 5 µg/mL, and AFWE is *A. vera* flower water extracts at 1, 2.5, 5 µg/mL. Treatment was carried out in normal condition. Each value represents the mean ± SEM of triplicate experiments. (*) *p* < 0.05 and (**) *p* < 0.01, (***) *p* < 0.001 compared to control group and (#) *p* < 0.05 and (##) *p* < 0.01, (###) *p* < 0.001 compared to positive control group.

HaCaT cells treated with AFWE under normal conditions showed no signs of cellular toxicity compared to the control or positive control groups as shown (Figure 1A). At three different concentrations (1, 2.5, and 5 µg/mL), AFWE significantly upregulated IVL protein expression in a dose-dependent manner (Figure 1C,D). The obtained results from ELISA demonstrated that the hyaluronan secretion was also increased by AFWE (Figure 1B). The expression of HAS1 by AFWE was increased in a concentration-dependent way even more than that in the AFWE treated control and positive control group. However, HYAL1 was decreased by AFWE in a comparison with the positive control group (Figure 1C,D).

### 2.2. AFWE Induces FLG Formation and AQP3 Expression

**Figure 2:**
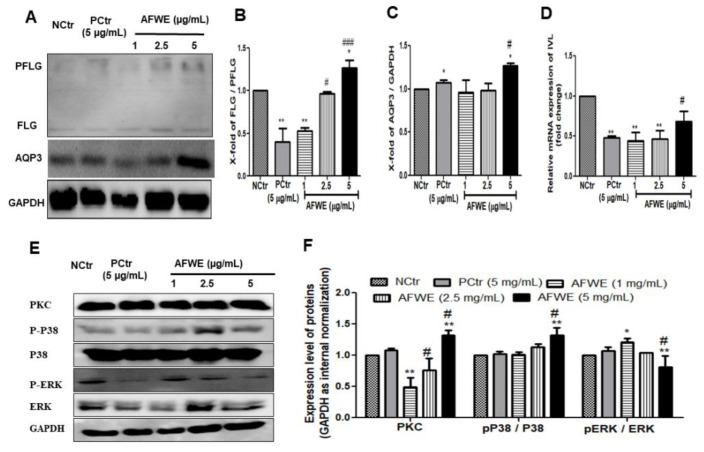
AFWE improves the expression of skin barrier function-related proteins on HaCaT cells. (**A**) Western blot analysis of FLG and AQP3 protein expression. (**B**) Fold change of PFLG to FLG ratio. (**C**) Fold change of AQP3 protein expression compared to control group. (**D**) Relative mRNA expression of IVL checked by qRT-PCR. (**E**) Western blot analysis of PKC and MAPK (pP38/p38, ERK 1/2) signaling pathway-related proteins expression. (**F**) Fold change of PKC, MAPK (pP38, ERK 1/2) signaling pathway-related proteins compared to control group. NCtr is control, PCtr is *C. asiatica*, used as a positive control at 5 µg/mL, and AFWE is *A. vera* flower water extracts at 1, 2.5, 5 µg/mL. Treatment was carried out in normal condition. Each value represents the mean ± SEM of triplicate experiments. (*) *p* < 0.05 and (**) *p* < 0.01, compared to control group and (#) *p* < 0.05 and (###) *p* < 0.001 compared to positive control group.

AFWE treatment group induced FLG expression compared to both the control and positive control group (Figure 2A,B). Furthermore, it also increased the expression of AQP3, another moisture-associated epidermal protein, in a dose-dependent manner (Figure 2A,C).

### 2.3. AFWE Regulates IVL Expression via Activation of PKC and MAPK Signaling Pathway

The western blot analysis demonstrated that AFWE significantly increased IVL protein expression dose-dependently (Figure 1C). Furthermore, qRT-PCR analysis was applied to elucidate the effects of AFWE on the mRNA level of IVL. Though there were no remarkable effects were observed (Figure 2D). 

For the regulation of IVL, activation of PKC, and different differentiation markers including P38 and ERK1/2 of MAPK signaling pathways play a vital role. In this current study, AFWE treatment increased the expression of PKC compared to the control and positive control group in HaCaT cells. AFWE also demonstrated to increase in phosphorylation of P38 and slight dephosphorylation of ERK 1/2 protein expression as compared to the control group (Figure 2E,F).

### 2.4. HPLC Analysis Confirms the Presence of Active Constituents of AFWE Extract

**Figure 3:**
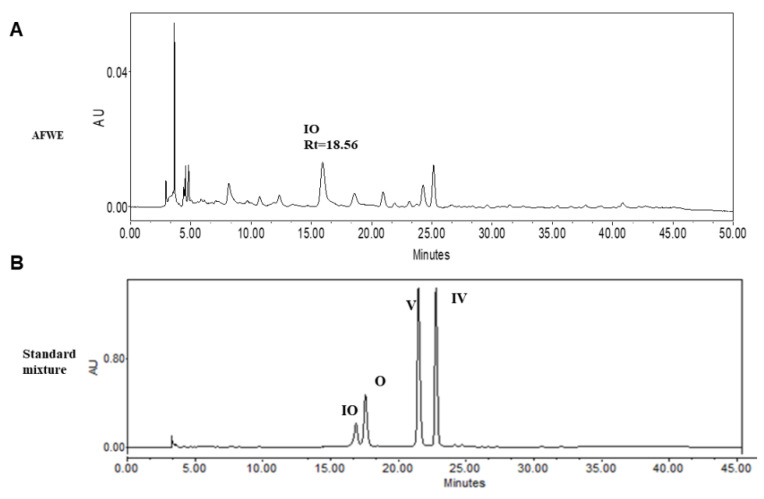
HPLC chromatogram of AFWE extract along with its major constituents. (**A**) HPLC chromatogram of AFWE extract (**B**) Chromatogram of standard compounds: IO, O, V and IV.

TLC and HPLC analysis confirmed the presence of IO, V, and IV in AFWE. Furthermore, among these constituents, the concentration of IO was higher than other constituents as shown in supplementary materials, Figure S2B (TLC), and Figure 3. The yield for AFWE was 22.24% and the content analysis indicated the presence of the phytoconstituents in the descending order: IO > V > IV mentioned in Table 1. Then, IO was taken into account as active constituents and further experiments are procced for moisturizing effects of IO.

### 2.5. Isoorietin (IO), an Active Constituent of AFWE Demonstrates Moisturizing Effects

**Figure 4:**
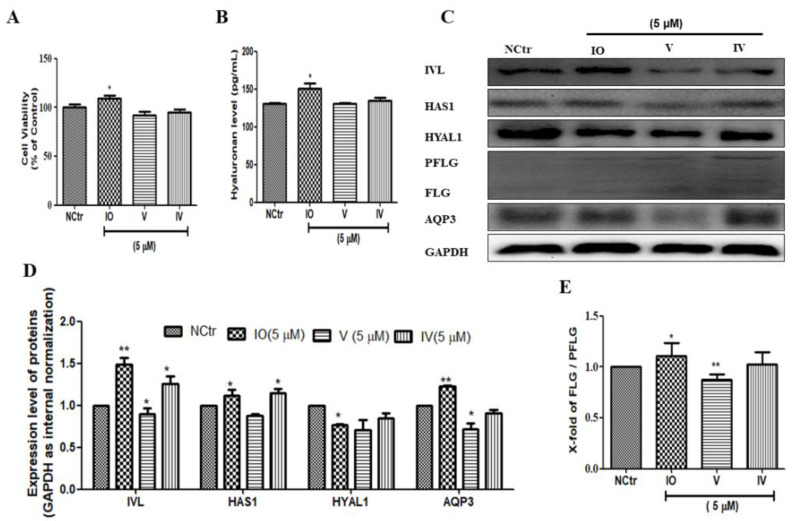
Isoorietin (IO), an active constituent of AFWE demonstrates moisturizing effects. (**A**) Cell viability of IO, V, and IV by MTT. (**B**) Hyaluronan content detected by ELISA. (**C**) Western blot analysis of IVL, HAS1, HYAL1, FLG, and AQP3, etc. expression. (**D**,**E**) Fold change of IVL, HAS1, HYAL1, AQP3, and FLG expression compared to the control group. NCtr is control, IO; Isoorientin, V; Vitexin, IV; Isovitexin. All of them were treated at 5 µM concentration. Treatment was carried out in normal condition. Each value represents the mean ± SEM of triplicate experiments. (*) *p* < 0.05 and (**) *p* < 0.01 compared to control group.

To investigate the role of the active constituents present in AFWE, we checked all the moisturization-related proteins using the major constituents. Cell viability was not significantly different in IO, V, or IV (5 μM) treated groups compared with that in the control group (Figure 4A). Among others, IO upregulated IVL, as well as increased hyaluronan content (Figure 4B), HAS1, and AQP3 expression. Additionally, IO slightly upregulated the expression level of FLG (Figure 4E) and downregulated the HYAL1 (Figure 4C,D). From the above results, it is clear that IO is the most active compound among others and it demonstrates skin moisturization effects targeting IVL expression.

### 2.6. Isoorietin (IO) Also Activates PKC and MAPK Signaling Pathway

**Figure 5:**
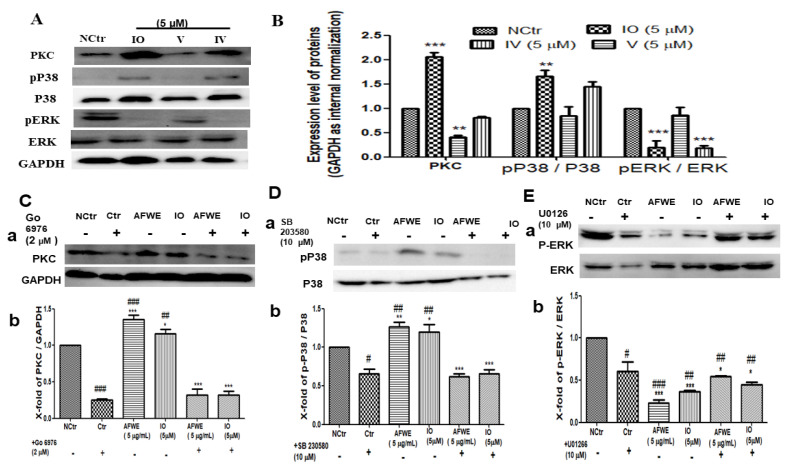
AFWE and IO both confirm the regulatory role of PKC and MAPK signaling in IVL expression. (**A**) Western blot analysis of PKC and MAPK (pP38/p38, ERK 1/2) signaling pathway-related proteins expression. (**B**) Fold change of PKC, MAPK (pP38, ERK 1/2) signaling pathway-related proteins compared to control group. (**C**) Western blot analysis of PKC protein expression after treatment with PKC inhibitor (Go 6976, 2 μM) (**a**) and fold change (**b**) compared to control group. (**D**) Western blot analysis of P38 and p-P38 protein expression after treatment with P38 inhibitor (SB 203580, 10 μM) (**a**) and fold change (**b**) compared to control group. (**E**) Western blot analysis of ERK and p-ERK protein expression after treatment with ERK inhibitor (U0126, 10 μM) (**a**) and fold change (**b**) compared to the control group. NCtr is control, IO; Isoorientin (5 µm), and AFWE is *A. vera* flower water extracts at 5 µg/mL. Treatment was carried out in normal condition. Each value represents the mean ± SEM of triplicate experiments. (*) *p* < 0.05 and (**) *p* < 0.01, (***) *p* < 0.001 compared to inhibitor group and and (#) *p* < 0.05 and (##) *p* < 0.01, (###) *p* < 0.001 compared to control group.

IO was found to be active among other constituents and also induced IVL expression predominately among other targets (Figure 4C,D). So, further experiments were carried out to confirm its function in the regulation of IVL expression through the activation of PKC and MAPK pathway proteins. According to the findings, there was a 2-fold increase in PKC expression, a 1.5-fold increase in P38 expression, and significant dephosphorylation of the ERK protein (Figure 5A,B).

### 2.7. AFWE and IO Both Confirm the Regulatory Role of PKC and MAPK Signaling in IVL Expression

Phosphorylation of PKC and P38 plays a very important role in the regulation of IVL expression [19,20]. Our findings indicate that complete inhibition occurred after treatment with PKC and P38 inhibitors. Then, the expression of PKC (Figure 5C) and P38 (Figure 5D) was found to be increased by the treatment of AFWE and IO. Furthermore, co-treatment of AFWE and IO along with the respective inhibitors again show the suppression of the respective protein expression. It is already reported that dephosphorylation of ERK is necessary for IVL expression [21]. However, AFWE and IO were found to suppress ERK expression even more than the ERK inhibitor group, and co-treatment of AFWE and IO with inhibitors upregulated the respective protein expression again (Figure 5E). Thus, it is obvious that the activation of PKC, P38, and deactivation of ERK are all known to regulate the IVL expression.

### 2.8. AFWE Protects UVB-Induced Photodamage in a Pattern Similar to That Observed in Normal Condition

**Figure 6:**
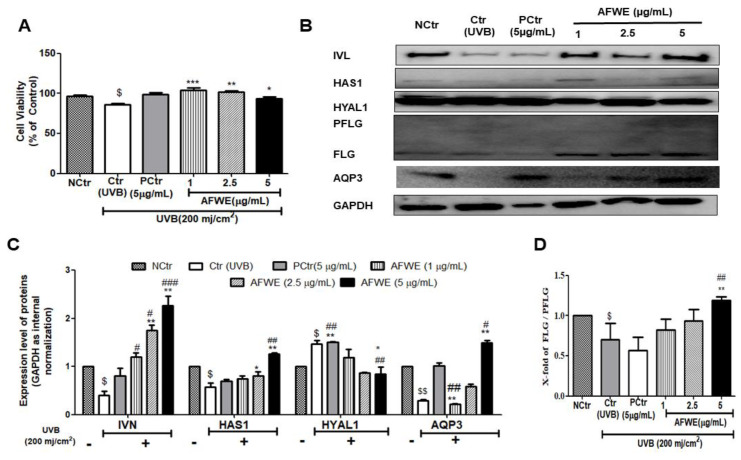
AFWE protects UVB-induced photodamage and restores skin hydration to the normal level. Post-treatment with AFWE was carried out on HaCaT cells with or without exposure to 200 mj/cm^2^ ultraviolet (UVB). (**A**) Cell viability of AFWE by MTT. (**B**) Western blot analysis of AFWE on IVL, HAS1, HYAL1, FLG and AQP3 expression (**C**,**D**) Fold change of of IVL, HAS1, HYAL1, AQP3 and FLG expression compared to control group. NC is normal control, Ctr is UVB control, AFWE is Aloe flower water extracts at 1, 2.5, 5 µg/mL concentration and CN is *Centella asiatica*, used as positive control at 5 µg/mL with or without exposure to 200 mj/cm^2^ ultraviolet (UVB). Each value represents the mean ± SEM of triplicate experiments. (*) *p* < 0.05 and (**) *p* < 0.01, (***) *p* < 0.001 compared to UVB control group; (#) *p* < 0.05 and (##) *p* < 0.01, (###) *p* < 0.001 compared to positive control group; and ($) *p* < 0.05 and ($$) *p* < 0.01 compared to normal control group.

UVB exposure causes photoaging, wrinkling, and pigmentation [22]. The UVB-irradiated group treated with AFWE showed no alteration in cellular morphology (Figure S2C), and no cell death was seen in the control or positive control groups (Figure 6A). Interestingly, AFWE treatment increased IVL expression in a dose-dependent manner, similar to that observed in normal conditions. Furthermore, it increased the expression of HAS1 as well as decreased the expression of HYAL1. Additionally, a significant dose-dependent increase in the expression of FLG protein was observed in the presence of AFWE. Furthermore, an increase in AQP3 expression was observed in UVB exposed AFWE treated cells (Figure 6B–D).

### 2.9. Molecular Docking Studies

**Figure 7:**
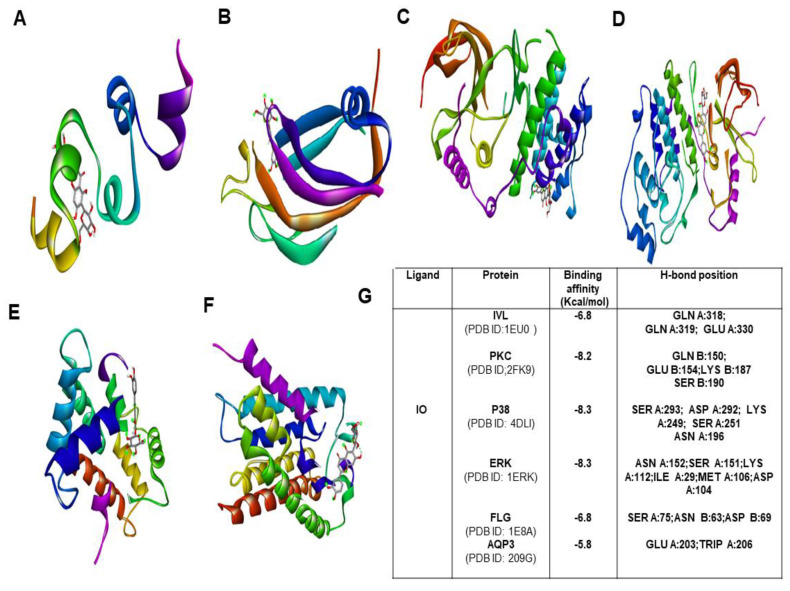
Molecular docking study of IO with various moisturization-related proteins performed by auto dock vina. The complex structure of IO with (**A**) IVL (**B**) PKC (**C**) P38 (**D**) ERK 1 (**E**) FLG (**F**) AQP3 (**G**) list of docking scores of the complex form (**A**–**F**).

**Figure 8:**
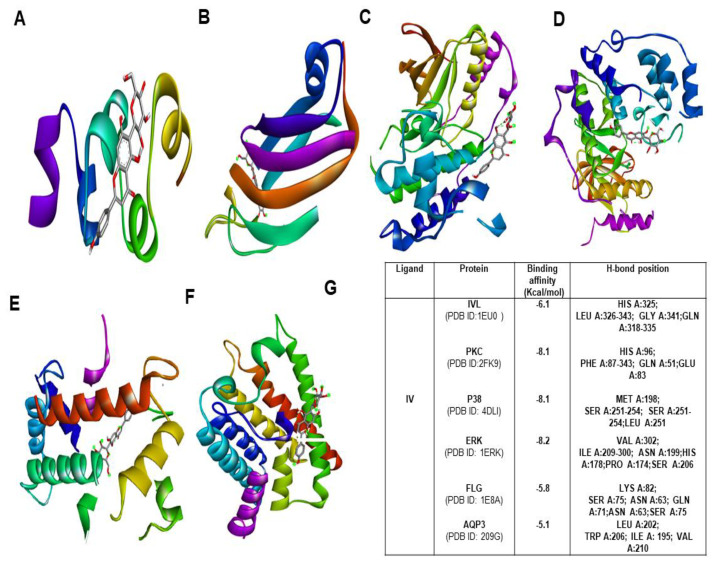
Molecular docking study of IV with various moisturization-related proteins performed by auto dock vina. The complex structure of IV with (**A**) IVL (**B**) PKC (**C**) P38 (**D**) ERK 1 (**E**) FLG (**F**) AQP3 (**G**) list of docking scores of the complex form (**A**–**F**).

To explore the binding affinity of the major active phytoconstituent of *Aloe vera* flower, molecular docking of IO and IV with all of its targets was performed. The results showed strong binding affinities (Figure 7G) with IO as evident from the binding score of closer to −10 (kcal/mol) (Figure 7; Figures S2–S5). IO showed strong binding affinities towards PKC, P38, and IVL. In the case of IV, the obtained docking score was less than the docking score of IO (Figure 8A–G).

## 3. Discussion

Moisturizers are always recommended to treat dry skin and its associated disorders. Among different natural medicinal plants, *Aloe vera* and its flowers have been used to exert emollient and humectant effects in beauty products and cosmeceuticals from the ancient periods [14,18]. However, there are no reports on the skin moisturization effects of the flower parts of *Aloe vera*. Therefore, this study has been aimed to explore the moisturization effects and its underlying molecular mechanisms. of *Aloe vera* flower along with its active constituents. In achieving our aims, we first conducted the skin moisturization effects by using three different types of *Aloe vera* flower extracts namely water (AFWE), 100% ethanol (AE), and 50% ethanol (EE) respectively. Based on our data, we found that among three different types of extracts, AFWE showed the highest moisturizing effects both in normal and UVB irradiated conditions. More specifically, its active constituent IO has indicated a promising effect compared with the others, possibly due to its abundance. *C. asiatica*, a common plant used in cosmeceuticals for its anti-inflammatory, wound healing as well as skin hydration effects [23]. The ethanolic extract of this plant and also its isolated compound, madecassoside, showed notable skin moisturization effects in a recent study [24]. Therefore, we utilized *C. asiatica* extract as a positive control to compare the effects of AFWE. Though we didn’t check and compare the impacts of IO with madecassoside in this investigation which can be confirmed in further study.

Skin moisturization relies solely upon the epidermal moisturization-related components. Preliminary data indicated among the three different extracts of *A. vera* flower (AFWE, EE and AE), AFWE was the most potential (Figure S1). Further experiments are based on the dose-dependent effect of AFWE. Based on our data, AFWE-treatment increases the HA level in HaCaT cells by increasing HAS1 expression and decreasing HYAL1 expression. Also, AFWE enhanced AQP3 expression. However, around 1.5-fold increase of IVL expression was noted. Taken together revealing the significant moisturization effect of AFWE targeting IVL potentially.

Epidermal differentiation is crusial for skin hydration and barrier function. Keratinocytes of the epidermis differentiate and migrate to SC which contains corneocytes. These corneocytes are enriched with NMF and IVL and FLG, the representative genes of keratinocyte differentiation markers [25]. Our studies explored that AFWE promotes degradation of PFLG to FLG to produce NMF significantly higher in comparison to control and positive control groups. Interestingly, IVL was also found to be modulated by a 2-fold increase. However, the mRNA expression level was not seen to be modified. This reveals that AFWE improves skin moisturization and barrier function targeting IVL significantly. Differentiation and maturation of epidermal keratinocytes require the phosphorylation of PKC [26], P38, and dephosphorylation of ERK1/2, in turn, increases the expression of IVL [27]. Our results indicated that AFWE-treatment induces approximately 1.5 times phosphorylation of P38 and dephosphorylation of ERK1/2 along with the activation of PKC in comparison with both control and positive control. However, the previous study reported that the IVL expression is regulated by the P38-ERK1/2 complex along with other factors [28]. Although we did not confirm the interaction of P38 and ERK1/2 in a complex form in this study that can be explored in the future.

Among different C-glycosyl flavonoids, IO is already reported to have numerous therapeutic effects, including anti-inflammatory, antioxidant effects, etc. Recently its role in UVB-induced skin injury has been reported [29]. However, little information is reported about its function in skin moisturization. The presence of active constituents was confirmed through the HPLC analysis. Interestingly, among all the constituents present in AFWE extract, IO was found to be the most abundant marker compound as shown in Figure 3. However, IO level in AE and EE was 392.9 ± 0.06 and 214.1 ± 0.09 ng/mg respectively which was comparatively less than the content in AFWE. IO was found with no cytotoxicity and causes 1.5-fold upregulation of IVL compared to the control group. Mentionable, the expression of HYAL1 was also seen to be reduced by IO. Thereafter, it was found to be effective among others by increasing the expression of HAS1, AQP3 and FLG. The obtained data indicated that IO might function as an active constituent and responsible for its moisturization effects on the *A. vera* flower targeting IVL potentially. Furthermore, activation of PKC and P38 as well as mentionable dephosphorylation of ERK protein was noted by the treatment of the IO group. Our findings show that IO is a major active constituent of AFWE, causing IVL expression to be induced through the PKC and MAPK signaling pathways, resulting in skin moisturization. Therefore, the use of inhibitors for PKC and MAPK pathway proteins was used to validate the findings.

In our study, after treatment of specific inhibitors for PKC and MAPK signaling pathway proteins (P38 and ERK) significant downregulation was noted. PKC and P38 protein expression were found to be restored subsequently after treatment with AFWE and IO. In the case of ERK, it was found with lower expression even more than the inhibitor treatment as well as control group, so phosphorylation of PKC and P38, as well as dephosphorylation of ERK is crucial step in the regulation of IVL protein influenced by AFWE and IO, as shown by these findings.

UVB exposure reduces the levels of hydration and moisture-related factors by raising SC thickness and disrupting SC barrier permeability [30]. In our study, the UVB-irradiated group was found to decrease with all the proteins associated with skin hydration and barrier function (HAS1, AQP3, FLG, IVL). However, after treatment with AFWE, the notable recovery of UVB irradiated damage was monitored. It substantially increased the expression of HAS1 in particular and decreased the expression of HYAL1. Moreover, it was found that the expression of AQP3 and filaggrin increased in a concentration-dependent way. Most importantly, there is a substantial upregulation of the expression of skin barrier function protein, IVL. Overall, a more than 2-fold increase is noticed in the case of HAS1, FLG and IVL expression by AFWE in UVB-induced conditions. All these factors function in line with the defensive moisturizing effects mediated by AFWE against UVB-induced conditions. However, the positive control group was unable to protect the UVB damage similarly by AFWE in terms of increasing all hydration and barrier-related protein.

In comparison to normal and UVB irradiation, AFWE was able to increase IVL, HAS1, filaggrin dose-dependently under both conditions compared to control and positive control groups. Interestingly, even after a severe decline in AQP3 expression via UVB exposure, it was able to increase significantly relative to the positive control group.

Over time, molecular docking studies are carried out to explore the protein and ligand interactions in a precise way [31]. From the obtained results, IO showed the highest docking score towards p38, ERK and PKC with a binding affinity −8.3 kcal/mol and −8.1 kcal/mol respectively. The second highest docking score was obtained with IVL and FLG (−6.8 kcal/mol). With AQP3, the least docking score (−5.8 kcal/mol) was formed. However, several intermolecular interactions including hydrogen bonds were involved which strongly defend the potential role of IO in skin hydration and skin barrier function. In the case of IV, the obtained docking score was highest (−8.2 kcal/mol) towards ERK and it shows the lowest binding affinity of −5.8 kcal/mol for AQP3.Most importantly, in a comparison of binding with IVL, IO showed a strong binding affinity with a docking score of −6.8 kcal/mol whereas IV has shown binging affinity of −6.1 kcal/mol only. Therefore it is also evident that the IO is the most potent active candidate than others for hydration-related proteins (Figure 7 and Figure 8).

Structurally, IO is a 6-C-glucosyl compound of luteolin, and V, IV is 8-C and 6-C-glucosyl derivatives of apigenin. Structural variations are responsible for the reported activities of flavonoids [32]. Since IO is structurally different from others and present to a greater extent. Therefore, it is obvious that it might be responsible for exerting skin moisturization effects of *Aloe vera* flower as active constituents and molecular docking scores are also assisted in our results. However, in vivo study as well as corresponding formulation design is required to further validate its claimed effects.

## 4. Materials and Methods

### 4.1. Chemicals and Reagents

Dried *Aloe vera* flower (UV-AVF1001) was a generous gift from Univera Co., Ltd. (Seoul, Korea). Dulbecco’s modified Eagle’s medium (DMEM), fetal bovine serum (FBS), and penicillin and streptomycin were purchased from Gibco-BRL (Grand Island, NY, USA). ELISA kit used for hyaluronan detection was purchased from R&D Systems Inc. (Minneapolis, MN, USA). PRO-PREP™ protein extraction solution, Enhanced chemiluminescence (ECL) detection kit was from Intron (Sungnam, Korea). Antibodies against GAPDH, IVL, HAS1, HYAL1, AQP3, Filaggrin, PKC, P38, ERK1/2, and secondary antibodies conjugated to horseradish peroxidase were purchased from Santa Cruz (Dallas, TX, USA), cell Science (Canton, MA, USA), and Cell Signaling Technology (Beverly, MA, USA). Inhibitors for PKC (Go 6976), P38 (SB 203580), ERK 1/2 (U0126) were purchased from Abcam and cell signaling technology, etc. All other chemicals were purchased from Sigma-Aldrich (Steinheim, Germany).

### 4.2. Extract Preparation

For three different extracts preparation, one gram of each dried and powdered *Aloe vera* flower was extracted with water (AFWE), 100% ethanol (AE), and 50% ethanol (EE) respectively. The extracts were then sonicated at 40 °C for 60 min followed by centrifugation and filtration. Then, the samples were evaporated using a rotary evaporator followed by freeze-drying. Finally, the extracts were stored at −4 °C until further use.

### 4.3. Cell Culture

HaCaT cells were obtained from the Korean Cell Line Bank (Seoul, Korea). The cells were cultured in high-glucose DMEM supplemented with 10% heat-inactivated FBS and 1% penicillin-streptomycin and incubated in 5% CO_2_ at 37 °C.

### 4.4. Sample Preparation and Treatment of Cells

The obtained three extracts were dissolved in PBS and filtered to make a stock of 10 mg/mL concentration respectively. Afterward, the stock solutions of each extract at 25 μg/mL and chemicals (IO, V and IV) at 5μM diluted in serum-free media were used for the treatment of the cells. Among three extracts, AFWE were treated further at 1, 2.5 or 5 μg/mL to evaluate concentration-dependent effects. HaCaT cells at a confluency of 80% were rinsed twice with PBS and then treated followed by incubation for 24 h. From different studies, it is already reported that *C. asiatica* can improve skin hydration [33,34] and we used this extract at 5 μg/mL as a positive control to compare the effects of AFWE.

UVB irradiation and treatment of HaCaT cells was performed according to a previously reported method [35] with slight modification at UVB (200 mj/cm^2^) by using a UVB irradiation machine (Bio-Link BLX-312; Vilber Lourmat GmbH, Marne-la-Vallée, France) followed by immediate treatment of previously prepared AFWE. Non-irradiated cells were considered as the negative control.

### 4.5. Cell Viability Assay

Cell viability was determined by MTT assay. HaCaT cells were seeded into 96-well plates (4 × 10^4^ cells/well) in the culture medium containing 10% FBS. After 24 h of incubation, the cells were treated with samples of desired concentration. Following 24 h incubation, media was removed and 0.5 mg/mL of MTT solution was added and incubated again at 37 °C for 1 h in 5% CO_2_. After that MTT was removed and 100 μL of DMSO was added to each well and absorbance was measured at 570 nm using a microplate reader (Molecular Devices E09090; San Francisco, CA, USA).

### 4.6. Enzyme Linked-Immuno Sorbent Assay (ELISA)

HaCaT cells were seeded into 96-well plates (3 × 10^4^ cells/well) and then treated with samples of desired concentration, or the vehicle and incubated for 24 h. After the incubation, the supernatant was collected and used for HA content detection using ELISA according to the manufacturer’s instructions.

### 4.7. Western Blotting

HaCaT cells were harvested, washed with cold PBS (1×), and lysed in PRO-PREP™, containing protease and phosphatase inhibitors. Then, the obtained protein was estimated by Bradford’s assay. Thirty μg of protein for each group was loaded and separated by 10% SDS-PAGE gel electrophoresis, then transferred to nitrocellulose filter membrane followed by blocking with 5% skim milk. Then, overnight incubation was done with primary antibodies against GAPDH, IVL, HAS1, HYAL1, AQP3, FLG, PKC, P38, ERK1/2 at 4 °C. then, the membranes were incubated with respective horseradish peroxidase-conjugated secondary antibodies and protein bands were visualized by using enhanced chemiluminescence reagent in the Chemi DocXRS+ imaging system (Bio-Rad, Hercules, CA, USA). Then, densitometric analysis was performed by using Image Master TM 17 2D Elite software, version 3.1 (Amersham Pharmacia Biotech, Piscataway, NJ, USA) [36].

To find out the regulatory roles of PKC and MAPK signaling pathway proteins on the expression of IVL, HaCaT cells were exposed in the presence or absence of inhibitors for PKC (Go 6976, 2 μM), P38 (SB 203580, 10 μM), ERK 1/2 (U0126, 10 μM) around 60 min followed by AFWE treatment (5 μg/mL) and IO (5 μM) for 24 h. Then, the cell extracts were subjected to western blot analysis to check their expression [37].

### 4.8. Quantitative Real Time-Reverse Transcription-Polymerase Chain Reaction (qRT-PCR)

Total RNA from HaCaT cells (6-well plates, 3 × 10^5^ cells/well) was isolated using an RNA extraction kit (KeyGEN BioTECH, Nanjing, China). cDNA was synthesized from total RNA, and qRT-PCR was performed using the PrimeScript RT reagent kit (Takara, Beijing, China), according to the manufacturer’s instructions. The primer sequences used for qRT-PCR were as follows: IVL, forward: 5′-GTGGGGGAGAGAGGGAATTA-3′; reverse, 5′-CTCACCTGAGGTTGGGATTG-3′; GAPDH, forward: 5′-TCCACTGGCGTCTT CACC-3′, reverse: 5′-GGCAGAGATGATGACCCTTTT-3′. The expression of IVL mRNA was normalized to that of GAPDH.

### 4.9. Thin-Layer Chromatography (TLC) and HPLC Analysis of Aloe vera Flower Extracts

TLC and HPLC were carried out to detect and analyze the constituents present in the extracts. TLC was carried out according to a previous method [38]. The HPLC analysis was performed using a Waters system (Waters Corp., Milford, MA, USA) equipped with separation modules (e2695) and a photodiode array detector following the previous method [39]. For content analysis, 10 mg/mL of each extract was separated. O, IO, V and IV were used as standard compounds at a concentration of 1 mg/mL.

### 4.10. Molecular Docking Studies

Molecular docking was performed with IO, IV, and all hydration-related proteins by using the AutoDock Vina version 1.1.2 software program to obtain possible confirmations and orientations for the ligand [40]. Briefly, for ligand preparation, the 2D SDF format of IO and IV was attained from the PubChem database (https://pubchem.ncbi.nlm.nih.gov/ (accessed on 20 October 2020)) of the chemical repository having PubChem CID 114,776 and 162,350 respectively. All the moisturization-related proteins (3D structures) were obtained from Protein Data Bank (PDB) (https://www.rcsb.org/ (accessed on 20 October 2020)) database in PDB format. PyRx software was used to generate a PDBQT file containing protein structure with hydrogens in all polar residues. Ligands were prepared rotatable by modifying all bonds. Lamarckian Genetic algorithm (LGA) method was used for protein and ligand fixed docking. Grid box with default grid spacing and put on the center of ligand to create docking site on the target protein. After finishing the docking search, the best conformation was selected having the lowest binding energy. The interactions between protein-ligand complex conformations were analyzed by hydrogen bonds as well as bond lengths by using Discovery Studio Visualizer 16.1.0.15350.

### 4.11. Statistical Analysis

One-way analysis of variance (ANOVA) was performed using GraphPad Prism 5 (GrahPad Software Inc., La Jolla, CA, USA) for comparing different treatment groups, and a *p*-value < 0.05 was considered as statistically significant. The results are presented as mean ± standard error of the mean (SEM) from three independent experiments.

## 5. Conclusions

In summary, this study showed that water-soluble extract of *Aloe vera* flower (AFWE), especially its active constituent isoorientin (IO), might enhance skin moisturization by upregulating the differentiation marker, IVL. Additionally, AFWE enhanced the expression of its particular target IVL by the activation of PKC and MAPK. Furthermore, it increased the expression of key moisturizing factors including AQP3, FLG and hyaluronan. Besides, we demonstrated that AFWE can inhibit UVB-irradiated skin damage in human keratinocytes. Based on these findings, we strongly suggest that the *Aloe vera* flower and its active constituents may be a good skin moisturizer targeting involucrin (IVL) expression.

## Figures and Tables

**Table 1:** Content analysis of major constituents of AFWE (ng/mg) by HPLC.

Sample Name	IO	V	IV
Retention time	18.56	23.64	25.004
Contents in AFWE	518.7 ± 0.06	153.2 ± 0.9	264.8 ± 0.005

## Data Availability

The data presented in this study are available on request from corresponding author.

## Supplementary Materials

The following are available online. Figure S1. Effects of three different extracts (AE, EE, and AFWE) of *Aloe vera* flower on skin hydration; Figure S2. TLC and molecular docking study results; Figure S3. Molecular docking study results showing the pocket binding view of IO with PKC and P38.; Figure S4. Molecular docking study results showing pocket binding view IO with ERK and FLG; Figure S5. Molecular docking study results showing pocket binding view(a) and different types of bond found during complex formation(b) of IO with AQP3 (A) and HYAL1(B); Figure S6: Molecular docking study results showing pocket binding view(A) and different types of bond found during complex formation(B) of IV with IVL; Table S1. Content analysis of major constituents of EE and AE (ng/mg) by HPLC.

## Abbreviations

AFWE	*A. vera* flower water extracts
HaCaT cells	human epidermal keratinocytes
IO	Isoorientin
HPLC	High-performance liquid chromatography
IVL	Involucrin
Aquaporins	AQP3
HA	Hyaluronic acid
PKC	Protein kinase C
UVB	Ultraviolet B
ELISAs	Enzyme-Linked Immunosorbent Assays
