# Supplementary material for: Effects of Aloe vera Flower Extract and Its Active Constituent Isoorientin on Skin Moisturization via Regulating Involucrin Expression: In Vitro and Molecular Docking Studies
Source: Molecules. 2021 Apr 30;26(9):2626. doi: 10.3390/molecules26092626 (PMC8125160; doi:10.3390/molecules26092626)
Supplement: Supplementary file 1 [file molecules-26-02626-s001.zip › Supplementary material.pdf]

Figure S1: Molecular docking study results showing pocket binding view(A) and different types of bond found during complex formation(B) of IV with IVL.

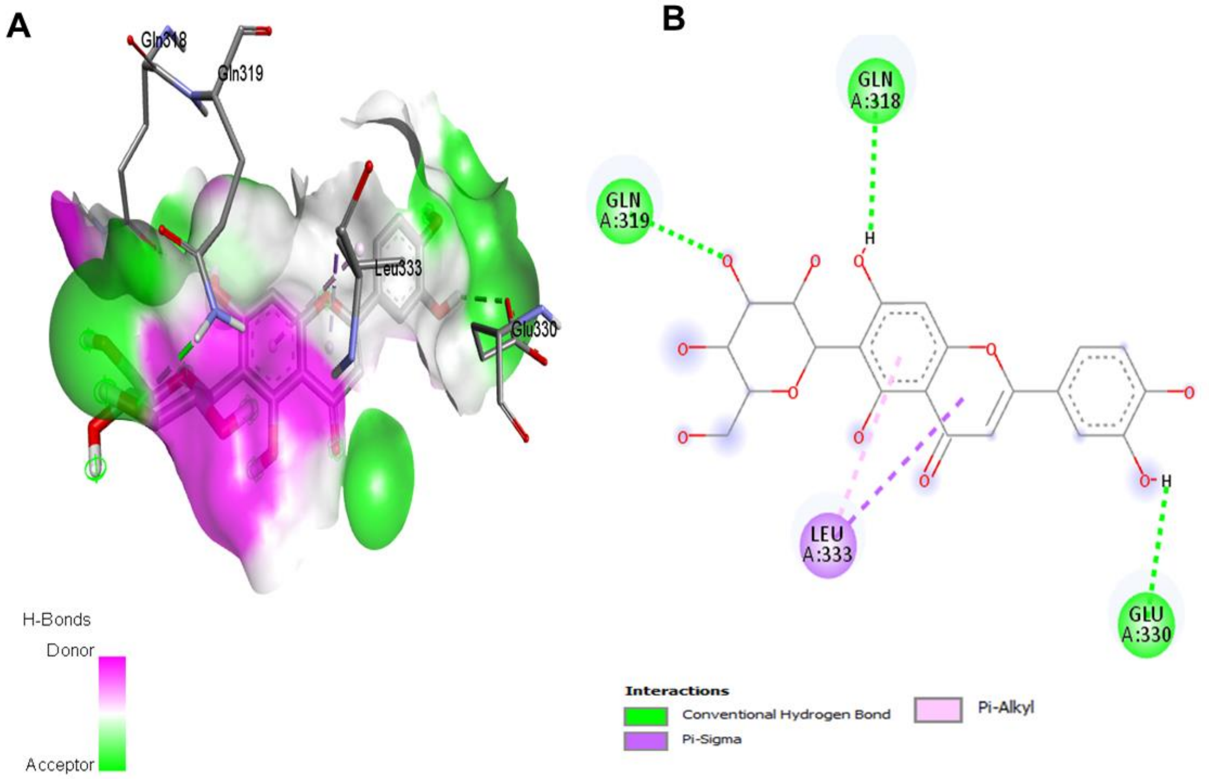

Table S1. Content analysis of major constituents of EE and AE (ng/mg) by HPLC.

**Sample Name**	**IO**	**V**	**IV**
**Retention time(Minutes)**	18.56	23.64	25.004
**Contents in EE**	392.99±0.062	118.10±0.069	224.12±0.002
**Contents in AE**	214.11±0.096	40.74±0.037	126.36±0.082
