# Supplementary figures and images for: Effects of Aloe vera Flower Extract and Its Active Constituent Isoorientin on Skin Moisturization via Regulating Involucrin Expression: In Vitro and Molecular Docking Studies
Source: Molecules. 2021 Apr 30;26(9):2626. doi: 10.3390/molecules26092626 (PMC8125160; doi:10.3390/molecules26092626)

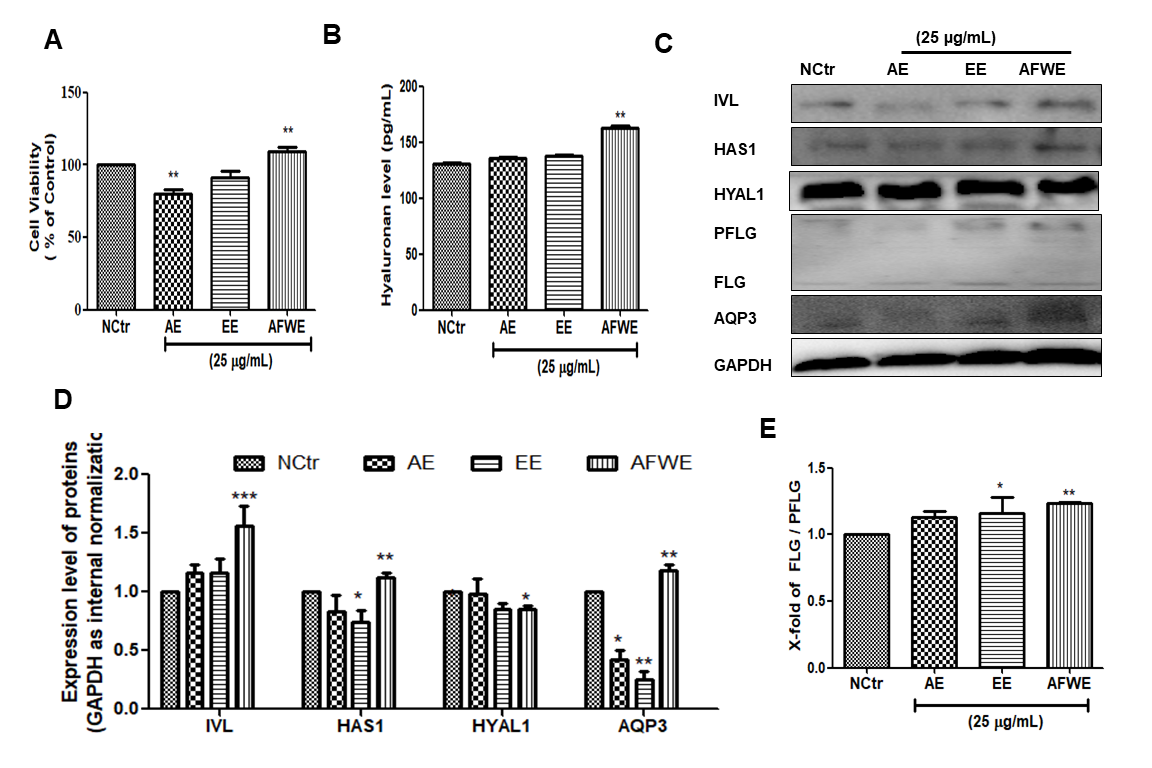

Supplement: Supplementary file 1 [file molecules-26-02626-s001.zip › Figure A1.TIF]

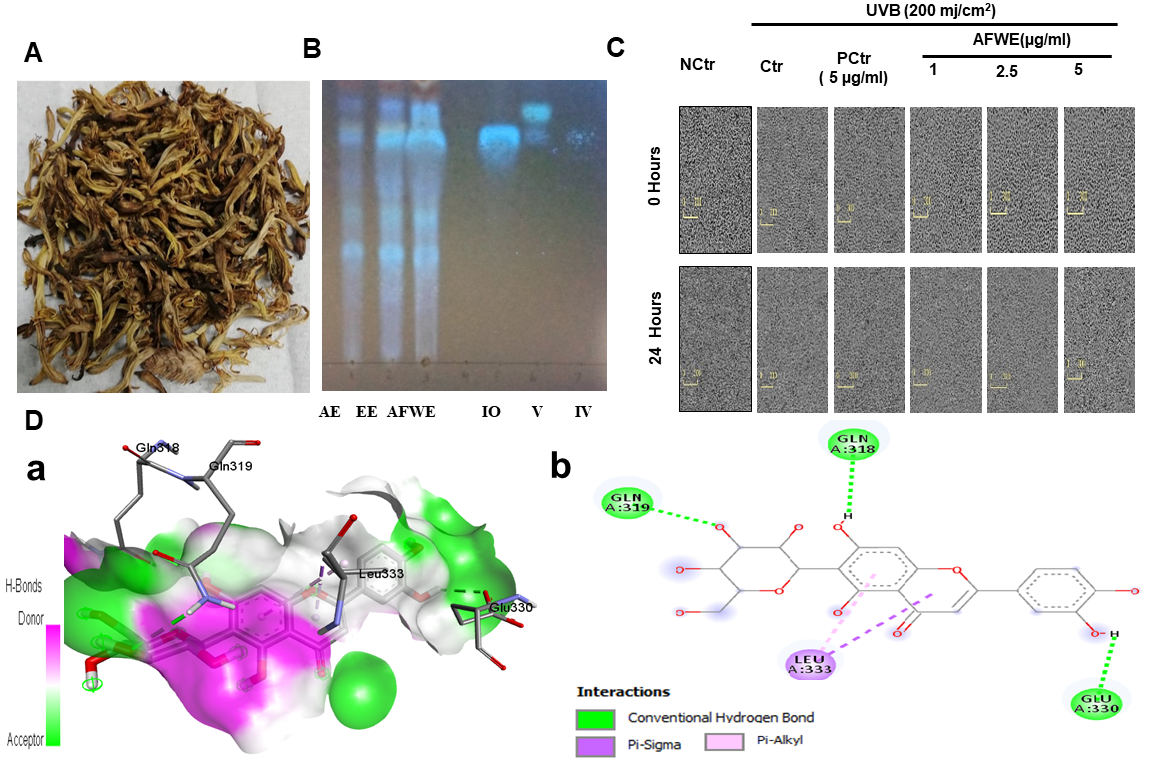

Supplement: Supplementary file 1 [file molecules-26-02626-s001.zip › Figure A2.TIF]

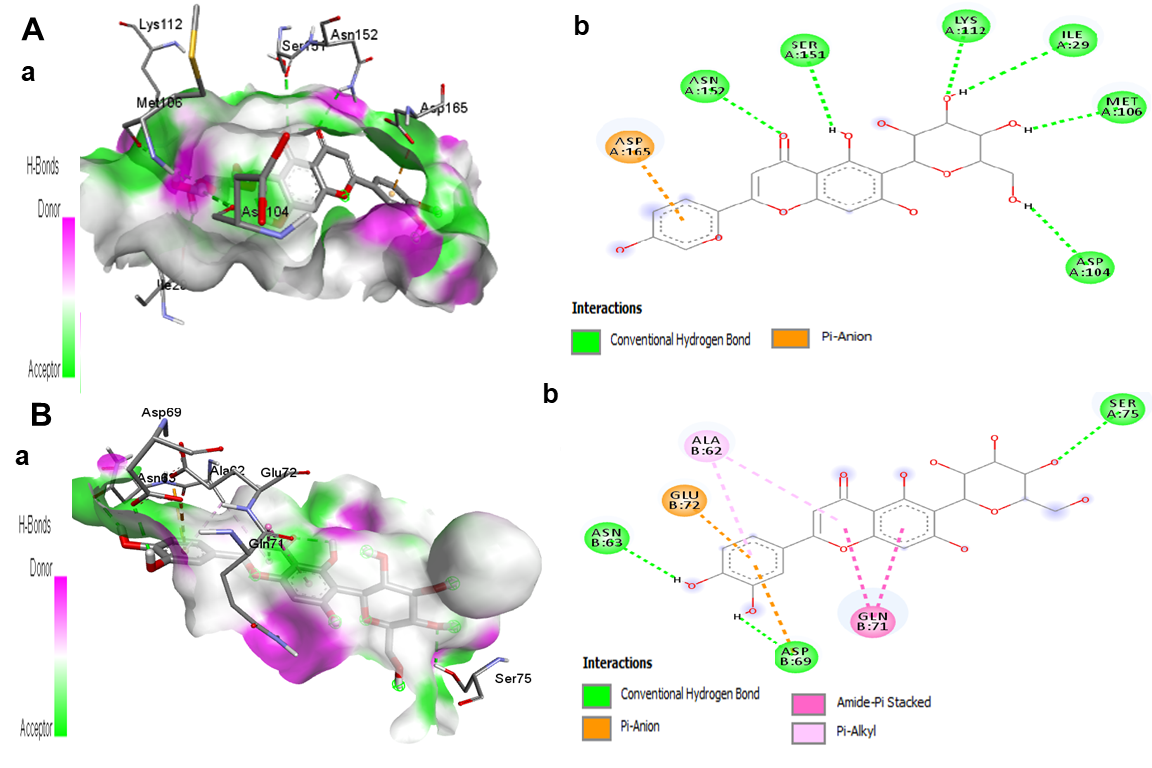

Supplement: Supplementary file 1 [file molecules-26-02626-s001.zip › Figure A3.TIF]

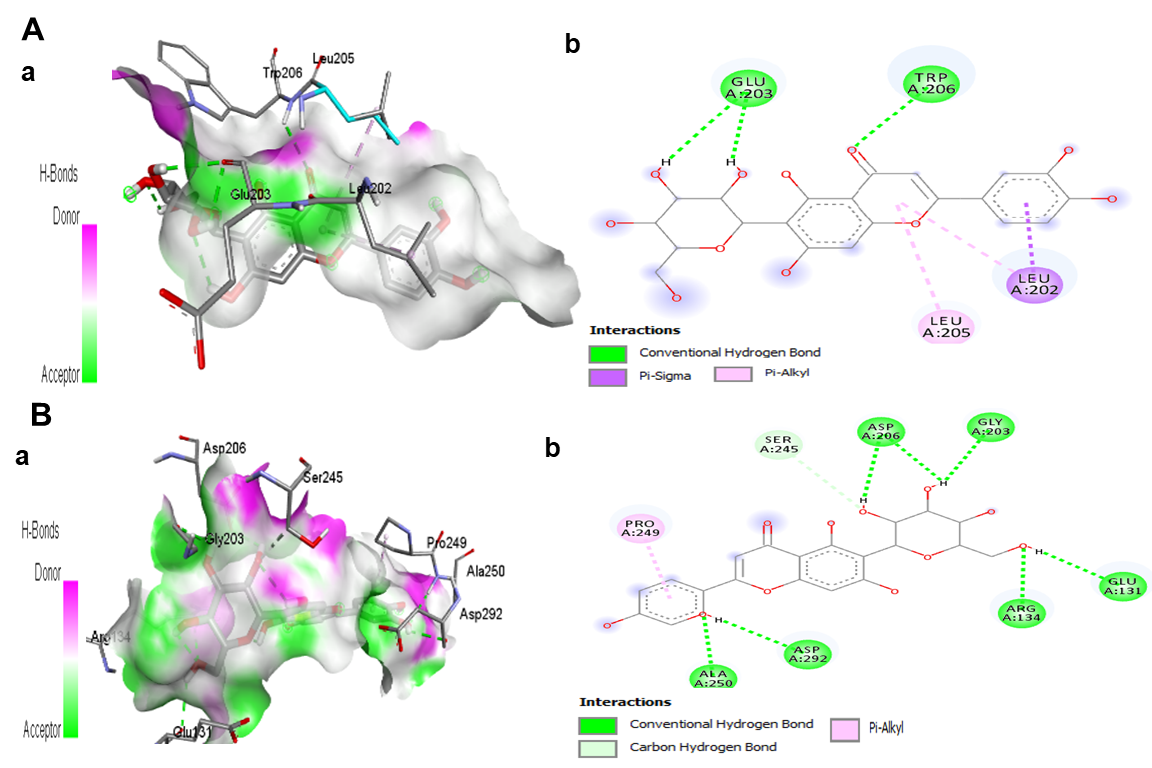

Supplement: Supplementary file 1 [file molecules-26-02626-s001.zip › Figure A4.TIF]

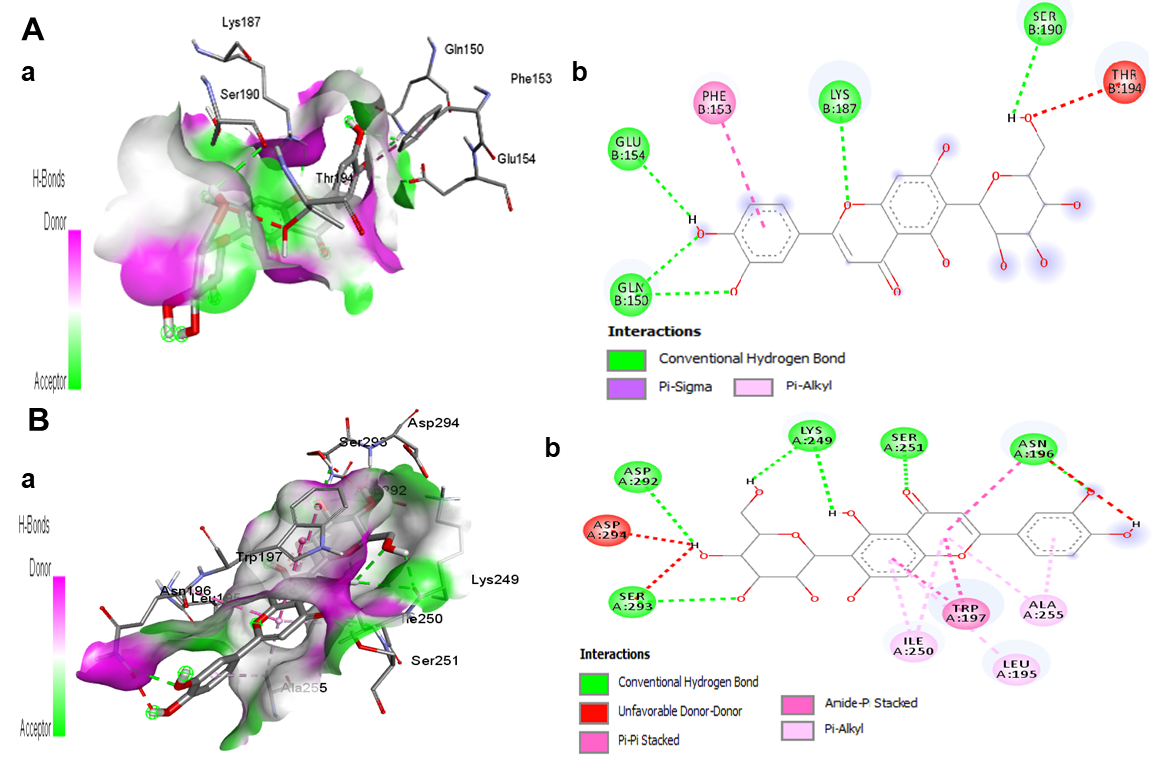

Supplement: Supplementary file 1 [file molecules-26-02626-s001.zip › Figure A5.TIF]
